# Performance enhancement of multiple-gate ZnO metal-oxide-semiconductor field-effect transistors fabricated using self-aligned and laser interference photolithography techniques

**DOI:** 10.1186/1556-276X-9-242

**Published:** 2014-05-17

**Authors:** Hsin-Ying Lee, Hung-Lin Huang, Chun-Yen Tseng

**Affiliations:** 1 Department of Photonics, Research Center Energy Technology and Strategy, Advanced Optoelectronic Technology Center, National Cheng Kung University, Tainan 701, Taiwan; 2 Institute of Microelectronics, Department of Electrical Engineering, Advanced Optoelectronic Technology Center, National Cheng Kung University, Tainan 701, Taiwan

**Keywords:** Laser interference photolithography, Multiple-gate metal-oxide-semiconductor field-effect transistors, Self-aligned photolithography, Short channel effect, Zinc oxide thin film

## Abstract

The simple self-aligned photolithography technique and laser interference photolithography technique were proposed and utilized to fabricate multiple-gate ZnO metal-oxide-semiconductor field-effect transistors (MOSFETs). Since the multiple-gate structure could improve the electrical field distribution along the ZnO channel, the performance of the ZnO MOSFETs could be enhanced. The performance of the multiple-gate ZnO MOSFETs was better than that of the conventional single-gate ZnO MOSFETs. The higher the drain-source saturation current (12.41 mA/mm), the higher the transconductance (5.35 mS/mm) and the lower the anomalous off-current (5.7 μA/mm) for the multiple-gate ZnO MOSFETs were obtained.

## Background

Over the past years, in view of the significant progress in fabrication techniques and epitaxial structures of III-V-based semiconductors [1-4], the III-V-based semiconductors were widely used in sensors [5,6], optoelectronic devices [7,8], electronic devices [9,10], and associated systems [11,12]. Among the electronic devices, the metal-oxide-semiconductor field-effect transistors (MOSFETs) are widely studied to improve the noise, output power, and power handling capacity [13,14]. Recently, because the ZnO-based semiconductors have the similar lattice constant and the same crystal structure with those of the GaN-based semiconductors, they make a promising potential candidate for replacing the GaN-based semiconductors due to their inherent properties including wide direct bandgap, large exciton binding energy, nontoxicity, stability, and biocompatibility. Several kinds of ZnO-based MOSFETs were reported, previously [15,16]. In general, single-gate structure was used to control the performances of the resulting MOSFETs. As predicated by the International Technology Roadmap for Semiconductors (ITRS), the dimension of the MOSFETs is continuously scaled down to reduce the area of integrated circuits. However, it becomes very difficult to maintain the necessary performances of the down-scaled MOSFETs owing to significantly short channel effects. To overcome the short channel effects, the architecture of double-gate (DG) MOSFETs [17], Fin FETs [18], HFin FETs [19], underlap FETs [20], and others was reported, previously. Compared with the single-gate MOSFETs, the peak lateral electrical field of the double-gate MOSFETs is lower [21]. Consequently, in addition to the suppression of the anomalous off-current caused by the field emission of carriers from channel defects, the gate length reduction is beneficial for enhancing the saturation current density and the transconductance of the resulting double-gate MOSFETs [22]. In this work, to study the channel transport control function of the multiple-gate structure, multiple-gate ZnO MOSFETs were fabricated and measured. Although the electron beam lithography is widely used to pattern narrow linewidth in devices, it suffers from high operation cost and complex equipment. In this work, the simple and inexpensive self-aligned photolithograph and laser interference photolithography were proposed to pattern the multiple-gate structure of the ZnO MOSFETs.

## Methods

**Figure 1:**
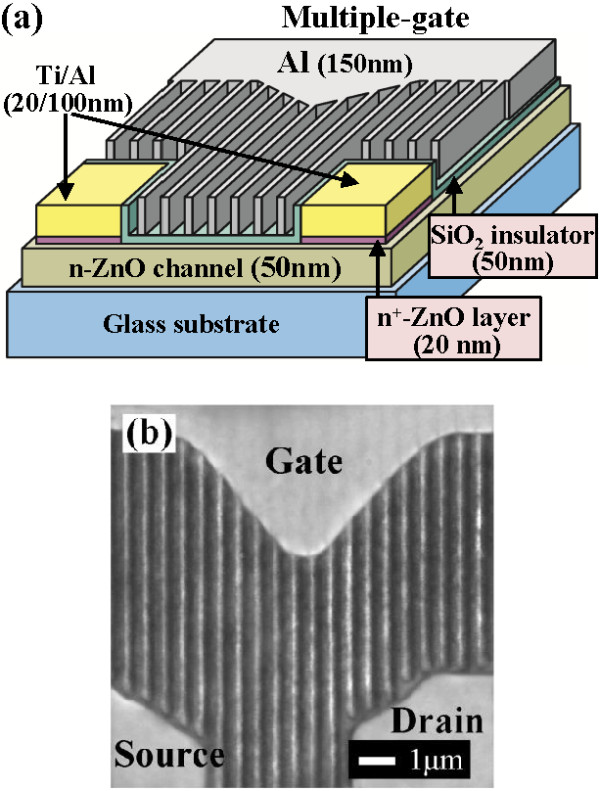
Schematic configuration (a) and SEM image (top view) (b) of multiple-gate ZnO MOSFETs.

**Figure 2:**
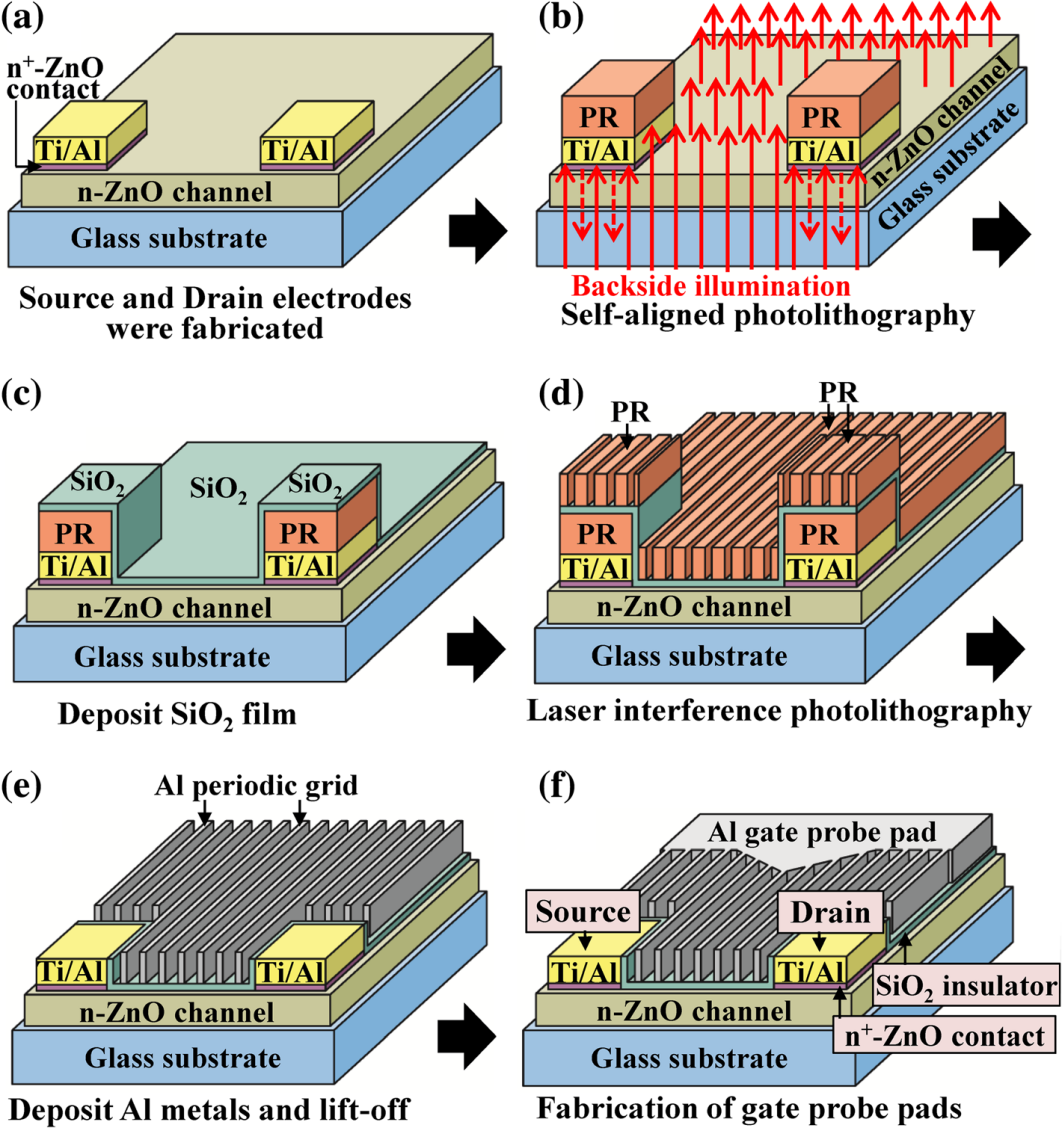
Fabrication processes (a to f) of multiple-gate ZnO MOSFETs using self-aligned photolithography technique and laser interference photolithography technique.

The schematic configuration of the multiple-gate ZnO MOSFETs and the scanning electron microscope (SEM) image of the multiple-gate structure are shown in Figure 1a,b, respectively. The mesa region was defined on the glass substrate using a standard photolithography technique. The ZnO target (purity = 99.99%, radio-frequency (RF) power = 100 W) and the Al target (purity = 99.99%, RF power = 15 W) were used as the material source for sputtering the 50-nm-thick Al-doped ZnO (ZnO:Al) film on glass substrates as the n-ZnO channel layer of ZnO MOSFETs. The n-ZnO channel layer was deposited using a radio-frequency magnetron co-sputter system under a working pressure of 30 mTorr and an Ar flow rate of 30 sccm. Using the Hall measurement at room temperature, the associated electron concentration and electron mobility of the n-ZnO channel layer were 3.5 × 10^17^ cm^−3^ and 9.7 cm^2^/V s, respectively. The mesa region was then formed using a lift-off process. After the source and drain regions were patterned using a standard photolithography technique, a 20-nm-thick n^+^-ZnO ohmic enhancement layer was deposited using ZnO target (purity = 99.99%, RF power = 100 W) and Al target (purity = 99.99%, RF power = 30 W) in the RF magnetron co-sputter system under a working pressure of 30 mTorr and an Ar flow rate of 30 sccm. The associated electron concentration and the electron mobility of the n^+^-ZnO ohmic enhancement layer were 4.1 × 10^19^ cm^−3^ and 3.6 cm^2^/V s, respectively. Ti/Al (20/100 nm) ohmic metals were then evaporated on the n^+^-ZnO region using an electron beam evaporator. Except for the source and drain regions, the excess n^+^-ZnO region and Ti/Al metal layers were removed using a lift-off process. To form ohmic contact, the sample was annealed in an N_2_ ambient at 200°C for 3 min. Figure 2 illustrates the fabrication process of the multiple-gate structure in this work. To avoid the source and drain regions being covered by the consecutively deposited SiO_2_ gate insulator, a positive photoresist (AZ6112) layer was patterned on the source and drain regions using a self-aligned technique. In the self-aligned technique, the sample was exposed from the backside illumination by using the mask of the source and drain metal electrodes. After a development process, only the photoresist layer residing on the source and drain electrodes was remained as shown in Figure 2b. A 50-nm-thick SiO_2_ gate insulator layer was then deposited using the RF magnetron sputter system under a working pressure of 10 mTorr and an Ar flow rate of 30 sccm as shown in Figure 2c. To prevent the source and drain electrodes from contacting with the subsequently deposited Al metal strips, before the process of the laser interference photolithography and the deposition of Al metal strips, the photoresist layer and the deposited SiO_2_ insulator layer residing on the source and drain electrodes were not removed instantly. After the deposition of the 50-nm-thick SiO_2_ insulator layer, the periodic strips of the multiple-gate structure were patterned using the laser interference photolithography technique. In the laser interference photolithography technique, the positive photoresist (Microposit S1818, Shipley, Marlborough, MA, USA) was firstly spread on the SiO_2_ insulator layer and then was exposed using two intersected He-Cd laser beams (power density = 0.7 mW/cm^2^ and wavelength = 325 nm) with a required interference fringe for 10 min. It is worthwhile to note that the SiO_2_ layer residing on the top and the side wall of the source and drain electrodes could protect the photoresist from being dissolved in the development process of the laser interference photolithography to insure the subsequent lift-off process. After the subsequent development procedure, a periodic photoresist strip pattern was defined as shown in Figure 2d. A 150-nm-thick Al gate metal layer was then evaporated using an electron beam evaporator. Using a standard lift-off procedure, the required Al gate strips with a strip width of 0.12 μm and a strip spacing of 0.42 μm were formed on the gate insulator layer; the unwanted part of the SiO_2_ insulator layer and the Al periodic strips residing on the source and drain electrodes were simultaneously removed as shown in Figure 2e. Finally, to fabricate multiple-gate ZnO MOSFETs, a 150-nm-thick Al gate probe pad was deposited and formed using a standard photolithography technique as shown in Figure 2f. The spacing between the source electrode and the drain electrode was 4 μm. There are seven gate strips between the source and drain metal electrodes in the resulting multiple-gate ZnO MOSFETs. Furthermore, to study for the channel transport control function of the multiple-gate structure, the conventional single-gate ZnO MOSFETs with a gate length of 1 μm were also fabricated and measured.

## Results and discussion

**Figure 3:**
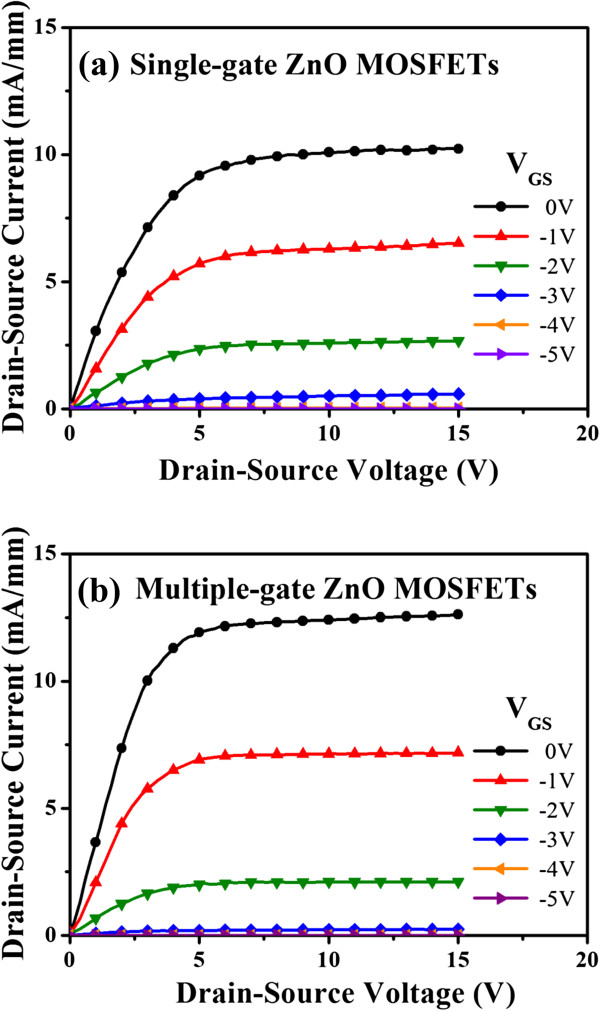
**Output characteristics of drain-source current.** As a function of drain-source voltage for **(a)** single-gate ZnO MOSFETs and **(b)** multiple-gate ZnO MOSFETs.

Figure 3a,b, respectively, shows the characteristics of the drain-source current (*I*_DS_) as a function of the drain-source voltage (*V*_DS_) of the single-gate ZnO MOSFETs and the multiple-gate ZnO MOSFETs measured using an Agilent 4156C semiconductor parameter analyzer (Santa Clara, CA, USA). The gate bias voltage (*V*_GS_) varied from 0 to −5 V in a step of −1 V. Compared with the single-gate ZnO MOSFETs, the drain-source saturation current (*I*_DSS_) of the multiple-gate ZnO MOSFETs operated at the same gate-source voltage = 0 V was improved from 10.09 to 12.41 mA/mm. The drain-source saturation current enhancement of the multiple-gate ZnO MOSFETs could be attributed to the reduction of the effective gate length. The length of the depletion region in the ZnO channel layer was commensurate with the gate length. Since the effective gate length of the multiple-gate structure was shorter than that of the single-gate structure, the series resistance between the source electrode and the drain electrode of the multiple-gate ZnO MOSFETs could be effectively reduced [22]. Moreover, the shorter source-gate distance in the multiple-gate ZnO MOSFETs could increase the electric field intensity along the ZnO channel between the source electrode and the gate electrode, in comparison with that of the single-gate ZnO MOSFETs. The increased electric field intensity could cause a higher electron velocity [23,24]. Therefore, the higher drain-source saturation current of the multiple-gate ZnO MOSFETs could be obtained.

**Figure 4:**
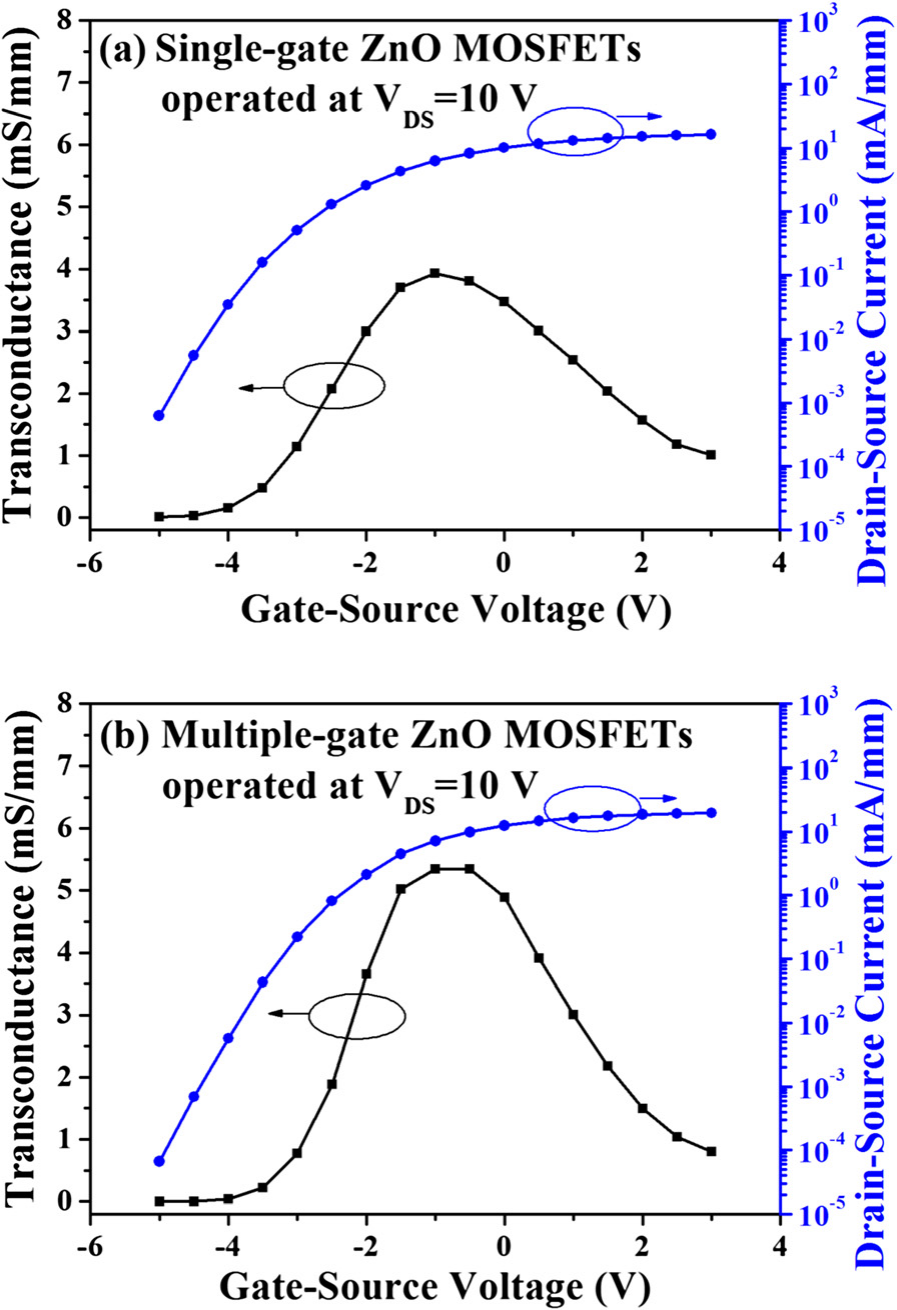
**Drain-source current and transconductance.** As a function of gate-source voltage for **(a)** single-gate ZnO MOSFETs and **(b)** multiple-gate ZnO MOSFETs.

Transconductance (*g*_m_), which is defined as the slope of the drain-source current as a function of the gate-source voltage, is an important parameter of MOSFETs. The dependence of the transconductance on the gate-source voltage of the single-gate ZnO MOSFETs and the multiple-gate ZnO MOSFETs operated at a drain-source voltage of 10 V was shown in Figure 4a,b, respectively. The maximal transconductance of the single-gate ZnO MOSFETs and the multiple-gate ZnO MOSFETs was 3.93 and 5.35 mS/mm, respectively. It could be found that the transconductance of the multiple-gate MOSFETs was higher than that of the single-gate ZnO MOSFETs. This result indicated that the multiple-gate structure exhibited better channel transport control capability. The transconductance in the saturated velocity model is inversely proportional to the depletion width [22]. Therefore, the multiple-gate ZnO MOSFETs with a shorter effective gate length could enhance the transconductance. Furthermore, the gate capacitance was increased by reducing the gate-source distance. The higher gate capacitance was also beneficial to an increase of the transconductance [24,25].

**Figure 5:**
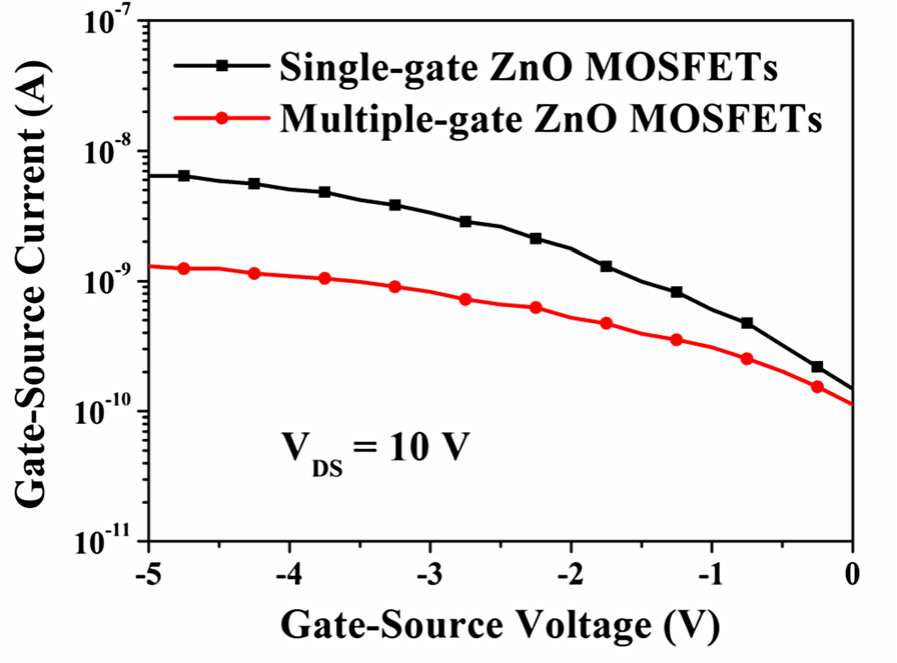
Gate-source current as a function of gate-source voltage for single-gate ZnO MOSFETs and multiple-gate ZnO MOSFETs.

In general, the gate-source electrical field (*E*_GS_) was relatively small in comparison with the gate-drain electrical field (*E*_GD_) since the gate-source voltage was smaller than the gate-drain voltage (*V*_GD_) [24]. The maximum gate-drain electrical field along the ZnO channel was located between the gate electrode and the drain electrode closed to the side of the gate electrode. It could be found that the gate-source electrical field enhancement was beneficial to the improvement of the drain-source current. In contrast, the larger maximum gate-drain electrical field was one reason of anomalous off-current. As shown in Figure 4, the anomalous off-current of the single-gate ZnO MOSFETs and the multiple-gate ZnO MOSFETs operated at a gate-source voltage of −4 V was 34 and 5.7 μA/mm, respectively. The off-current of the multiple-gate ZnO MOSFETs was lower than that of the single-gate ZnO MOSFETs. It could be expected that the multiple-gate structure had a lower maximum gate-drain electrical field as reported previously [21,24]. To further investigate the function of the multiple-gate structure, the characteristics of the gate-source current (*I*_GS_) as a function of the gate-source voltage of both the ZnO MOSFETs were measured at a drain-source voltage of 10 V; the measured results were shown in Figure 5. Based on the measured results, the gate-source current of the multiple-gate ZnO MOSFETs was reduced at the negative gate bias regime in comparison with that of the single-gate ZnO MOSFETs. The results revealed that the multiple-gate structure could disperse the gate surface carrier density due to the larger surface area with respect to the single-gate structure. The lower gate surface carrier density could effectively suppress the carrier injection opportunity from the gate electrode. Therefore, the gate-source current of the ZnO MOSFETs could be significantly improved by utilizing the multiple-gate structure.

## Conclusions

In conclusion, the self-aligned photolithography technique and the laser interference photolithography technique were used to fabricate the multiple-gate structure of multiple-gate ZnO MOSFETs. The multiple-gate structure had a shorter effective gate length and could enhance the gate-source electrical field and reduce the maximum gate-drain electrical field in comparison with the single-gate structure. Therefore, the performance of the multiple-gate ZnO MOSFETs was improved. Compared with the single-gate ZnO MOSFETs, the associated performances of the multiple-gate ZnO MOSFETs, including a higher drain-source saturation current of 12.41 mA/mm, a higher transconductance of 5.35 mS/mm, and a lower anomalous off-current of 5.7 μA/mm, could be effectively enhanced. The experimental results verified that the high-performance multiple-gate MOSFETs could be fabricated by the proposed simple and cheaper method. When the laser with a shorter wavelength was used in the laser interference photolithography, the multiple-gate MOSFETs with nanometer-order gate length could be expected by using this proposed technique.

## Competing interests

The authors declare that they have no competing interests.

## Authors' contributions

H-YL conceived the study and participated in its design and coordination. H-LH and C-YT carried out the experiments. H-YL, H-LH, and C-YT drafted the manuscript. All authors read and approved the final manuscript.
